# Identification, Geochemical Characterisation and Significance of Bitumen among the Grave Goods of the 7th Century Mound 1 Ship-Burial at Sutton Hoo (Suffolk, UK)

**DOI:** 10.1371/journal.pone.0166276

**Published:** 2016-12-01

**Authors:** Pauline Burger, Rebecca J. Stacey, Stephen A. Bowden, Marei Hacke, John Parnell

**Affiliations:** 1 Department of Scientific Research, The British Museum, London, United Kingdom; 2 Department of Geology and Petroleum Geology, University of Aberdeen, Aberdeen, United Kingdom; New York State Museum, UNITED STATES

## Abstract

The 7^th^ century ship-burial at Sutton Hoo is famous for the spectacular treasure discovered when it was first excavated in 1939. The finds include gold and garnet jewellery, silverware, coins and ceremonial armour of broad geographical provenance which make a vital contribution to understanding the political landscape of early medieval Northern Europe. Fragments of black organic material found scattered within the burial were originally identified as ‘Stockholm Tar’ and linked to waterproofing and maintenance of the ship. Here we present new scientific analyses undertaken to re-evaluate the nature and origin of these materials, leading to the identification of a previously unrecognised prestige material among the treasure: bitumen from the Middle East. Whether the bitumen was gifted as diplomatic gesture or acquired through trading links, its presence in the burial attests to the far-reaching network within which the elite of the region operated at this time. If the bitumen was worked into objects, either alone or in composite with other materials, then their significance within the burial would certainly have been strongly linked to their form or purpose. But the novelty of the material itself may have added to the exotic appeal. Archaeological finds of bitumen from this and earlier periods in Britain are extremely rare, despite the abundance of natural sources of bitumen within Great Britain. This find provides the first material evidence indicating that the extensively exploited Middle Eastern bitumen sources were traded northward beyond the Mediterranean to reach northern Europe and the British Isles.

## Introduction

The early medieval ship-burial at Sutton Hoo (Suffolk, UK), is one of the most significant archaeological discoveries ever made in Britain for the magnificence of its contents. The burial (Mound 1), located within a 7^th^ century AD cemetery containing some eighteen mounds including both inhumations and cremations, was first excavated in 1939 and both the site and the grave assemblage have since been subject to continued archaeological research [1].

No original timbers from the ship survived in the Mound 1 burial, but many details of the construction were retained in the stained sand and nearly all of the iron planking rivets remained in situ. The evidence indicated a 27.3 m long clinker-built boat, the beam maximising at 4.5 m, with nine strakes on each side and possibly rowed by up to forty oarsmen [2]. Repair patches visible on the hull, suggested that the ship had seen navigational use and had not been constructed especially for the burial [3] and tests with a half-scale replica have demonstrated that the vessel was especially suited for sailing in shallow rivers and along coasts [4]. To be deposited in the mound, the vessel must have been dragged some 700 m inland from the nearby river Deben, to a trench dug to hold her and then covered by an artificial mound after the funeral. Time, combined with the weight of the mound, compressed the grave’s content in the red-brown sand.

A dark rectangle corresponding to the ruined burial chamber built amidship was visible in the soil at the centre of the ship-burial. No human body was found and initially, it was suggested that the ship-burial was a cenotaph, but further experiments and chemical analyses conducted during the British Museum excavation campaign (1966–1971) evidenced phosphate enrichment of grave goods discovered in the west end of the burial, supporting the idea that a corpse had disappeared in the highly acidic soil conditions [3,5].

**Fig 1:**
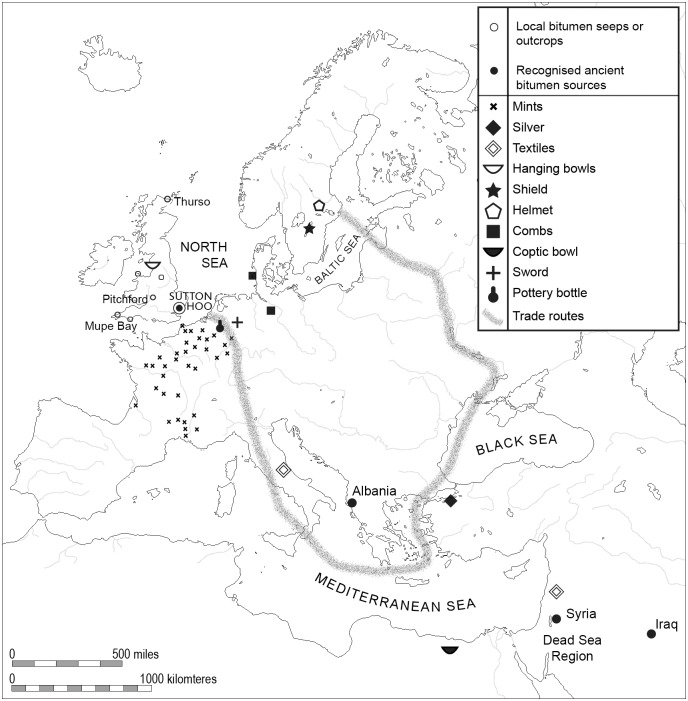
Map showing provenance of the grave goods found at the Sutton Hoo site (including the mints represented by the coinage) with major cross-continental trade routes indicated. Also marked are the locations of bitumen sources investigated during this study (adapted from Carver 2000 [3]).

Consistent with the traditions of its immediate northern and central European neighbours, e.g. the rite of ship-burial itself and the analogy of the Sutton Hoo helmet and shield with examples found in the contemporaneous Vendel and Valsgärde ship-burials from eastern Sweden [2, 6–8], the burial contained objects from throughout its contemporary known world (Fig 1), including coins minted in Merovingian France and Levantine textiles [2–3]. The richly furnished burial is commonly attributed to Raedwald (d. 624/5 AD), King of East Anglia and contains artefacts identified as gift exchange between East Anglian and foreign leaders (Fig 1) [2–3, 9–12]. The discovery of 12 surrounding burials which might be sacrificial burials, reinforce the interpretation of Mound 1 as a high-status grave. Together with the 6^th^ century Snape ship-burial discovered in 1862 at Snape Common, Suffolk, this site is of importance in understanding the role of the Kingdom of East-Anglia in the political landscape of early medieval Northern Europe and its connectivity with the wider world [2, 11, 13–14].

**Fig 2:**
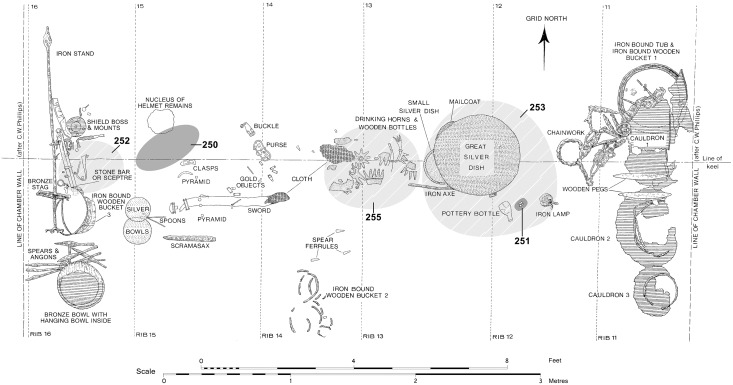
Plan of the Mound 1 burial chamber, combined from versions by Phillips [15] and Bruce-Mitford [17] with grey highlighted areas showing locations of the tarry finds listed in Table 1 by inventory number.

During the 1939 excavation, a number of groups of tarry-looking material were recorded within the burial chamber (Fig 2). Among these were two groups of three fragments located near the head and foot of the coffin. The location of the latter can be precisely identified because they were recorded on the first plan of the burial deposit, labelled as ‘manganese oxide’ based on unspecified analysis undertaken by F.E. Zeuner [15]. The fragments at the other end of the coffin were found close to the helmet remains, although their exact location was not reported. The six fragments are visually similar: each less than 20mm^3^, dark brown/black in colour and glossy with an irregular angular shape. Some faces are conchoidally fractured, others are smooth and flat and a few display scratches or impressions that may imply worked or moulded surfaces. Analysis of these fragments (by solubility tests and paper chromatography) in 1970 overturned the earlier manganese oxide identification [16] and described them as “Stockholm tar” produced by destructive distillation of wood from *Pinus sylvestris* L. [17]. Often used as a waterproofing agent and timber preservative, especially in maritime contexts [18–19], tar has been reported from other ship-burials, especially the 4^th^ century Slusegård boats (Denmark) [20] and the 6^th^ century Snape boat (England) [21]. Since their excavation, the tarry-looking lumps recovered from Sutton Hoo have resided in the collections of the British Museum (BM reg nos: 1939,1010.250/1); identified as “Stockholm tar” they were interpreted as repair materials, placed in the burial for maintenance of the ship in its afterlife voyage [3] or as surviving fillers from the lost timbers [1]. Other tarry materials from the ship marked in Fig 2, remained unidentified by the 1970 analysis, although Stockholm tar and manganese oxide were ruled out on the basis of solubility and elemental analysis [16].

Reinvestigation of the Sutton Hoo tars was undertaken within a wider research project examining the technology and preservation of ancient tars and pitches. The tar-like lumps were analysed by Fourier-transform infrared spectroscopy (FTIR), gas chromatography-mass spectrometry (GC-MS) and elemental analysis—isotope ratio mass spectrometry (EA-IRMS) and the surface morphology of the fragments was examined by optical microscopy and eflectance transformation imaging (RTI).

## Sampling and Analytical Methods

### Archaeological samples

**Fig 3:**
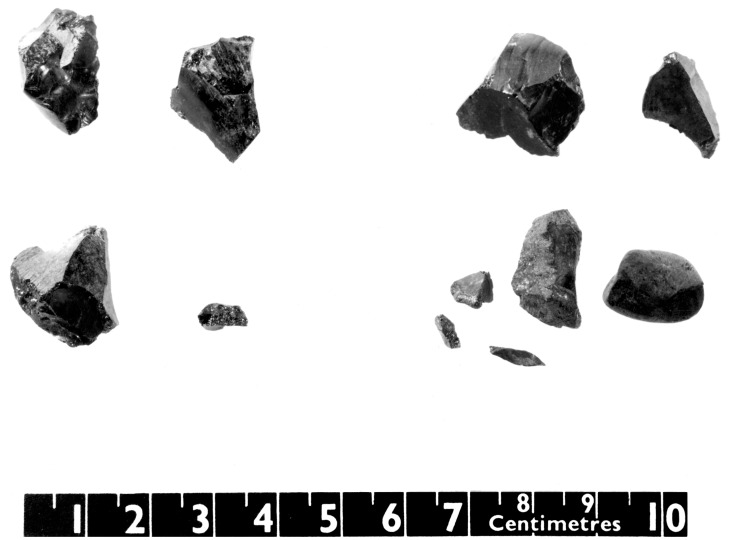
Some of the fragments from Sutton Hoo investigated in this study (BM registration numbers 1939.1010.250 and 1939.1010.251).

The various tarry lumps and fragments from the Sutton Hoo burial that were re-examined in this study are listed in Table 1. The primary focus of the work was the fragments that had been previously identified as tar (BM Reg nos.: 1939.1010.250 and 1939.1010.251; Fig 3); the other tarry-looking materials were included in case improved analytical technologies could shed further light on their character.

**Table 1:** Tarry finds from Mound 1 that were re-examined in this study.

BM Reg no. [Excavation inventory no.]	Description	Location in grave	Results of previous studies and/or interpretations	Analyses applied
1939.1010.250 [250]	Three black conchoidally fracturing lumps (with charcoal)	From near helmet	1939 -? pitch; 1940—manganese oxide; 1970—Stockholm tar; 1971 -? bitumen	FTIR; GCMS
1939.1010.251 [251]	Three black conchoidally fracturing lumps	From beside the pottery bottle (marked on Phillips’ 1940 plan)	1939 -? pitch; 1970—Stockholm tar; 1971 -? bitumen	FTIR; GCMS; EA-IRMS; RTI
1939.1010.252 [252]	Twenty conchoidally fracturing lumps, dull black surface but fresh breaks are shiny	Near shield rim, stone sceptre and bucket 3	1939 -? pitch; 1970—not Stockholm tar (according to label in box)	FTIR; GCMS
1939.1010.253 [253]	Two dark brown/black lumps with brittle slightly laminar structure	Near the large silver dish/ west end of coffin (originally boxed with silver rivets)	1939 -? pitch; 1970—not Stockholm tar	FTIR; GCMS
1939.1010.254 [254]	Tiny fragments of black glossy material, with wood and iron fragments	West end of chamber	1939 -? pitch; 1970—not Stockholm tar or manganese oxide	FTIR; GCMS
1939.1010.255 [255]	Two dark brown, glossy fragments with laminar structure. Very brittle	From drinking horn complex	1939 -? pitch; 1970—not seen	FTIR; GCMS

### Reference materials

To constrain the nature and origin of the Sutton Hoo tarry residues, their chemical composition was compared with a select group of British and Middle Eastern bitumen. Local (UK) specimens from on-shore petroleum systems included bitumen from Pitchford (Shropshire), Windy Knoll (Derbyshire), Great Orme’s Head (Gwynedd, Wales), South Crofty (Cornwall), Thurso (Caithness) and Mupe Bay (Dorset). Middle Eastern comparators originated from Syria, Lebanon and the Dead Sea region. See Fig 1 for locations.

### Analytical techniques

#### Reflectance transformation imaging

Surfaces of selected fragments (BM Reg nos.: 1939.1010.250 and 1939.1010.251) were imaged using an RTI (reflectance transformation imaging) dome system developed at the Electronics and Computer Science Department at the University of Southampton by Kirk Martinez and Philip Basford. The dome is a hemisphere of one metre diameter, with 76 LED lights (Bridgelux BXRA-56C1000-A-00 LEDs, with a colour temperature of 5600K) arranged inside four quadrants. Each LED is illuminated in turn, with a separate image of the object recorded each time. Images were recorded using a Nikon 800D camera positioned at the apex of the dome, giving a maximum field of view of 20 by 30 cm. The images were recorded using a procedure developed by Klaus Wagensonner [22]. The 76 images were compiled into the polynomial texture map (ptm) file using RTI Builder software, which was developed by Universidade do Minho and Cultural Heritage Imaging. The finished ptm files were viewed using RTIViewer, downloaded from the Cultural Heritage Imaging website (http://culturalheritageimaging.org/What_We_Offer/Downloads).

#### Fourier transform infrared spectroscopy

FTIR measurements were performed on a Nicolet 6700 spectrometer with Continuum IR microscope equipped with MCT/A detectors. Sub-milligram samples were analysed in transmission mode in a diamond microcompression cell (clean diamond window measured for background). The analysis area was controlled by the sliding aperture, maximising at 100x100 μm. Acquisition was achieved in the range 4000–650 cm^-1^ using 32 scans at a resolution of 4 cm^-1^ and automatic gain.

#### Gas chromatography-mass spectrometry

*Solvent extraction procedure*. For chromatographic analysis, *c*. 5 mg powdered samples were extracted with 200 μl dichloromethane (DCM). Prior to analysis, aliquots of 10 μl dried under nitrogen were derivatised with 50 μl bis(trimethylsilyl)trifluoroacetamide (BSTFA) and heated for 1 h at 70°C to form trimethylsilyl (TMS) derivatives.

*Fractionation procedure*. Fractionated extracts were prepared from *c*. 5–10 mg powdered sample extracted with 1 ml dichloromethane. The insoluble (inorganic) fraction was separated by settling. After evaporation under nitrogen, the extract was deasphalted four times by precipitation using DCM:Hexane 1:50 and centrifugation (10 min at 3500 rpm). The resulting maltene fraction obtained was sequentially fractionated on a silica gel gravity flow column (chromatography grade 60–120 μg silica gel, pre-extracted with DCM:methanol (MeOH) 97:3, followed by hexane and oven dried) using the following solvents: hexane for the saturated hydrocarbons, DCM:Hexane 1:3 for the aromatic hydrocarbons and DCM:MeOH 2:1 for the polar compounds. Fractions were dried under nitrogen and weighed.

*GC-MS analyses*. GC-MS analyses were performed on an Agilent 6890N gas chromatograph (GC) equipped with an HP5 column (30 m x 0.25 mm, 0.25 μm film thickness; Agilent 109091S-433) and coupled to an Agilent 5973N mass spectrometer (MS). Helium was used as carrier gas in constant flow (1.3 ml/min).

DCM extracts were injected splitless at 9.99 psi. After a 1 min isothermal hold at 35°C, the oven was temperature programmed to 300°C at 10°C/min with the final temperature held for 8 min. Acquisition was in scan mode (50–600 amu/sec) after a solvent delay of 7.5 min.

Saturated hydrocarbon fractions were injected splitless at 11.72 psi. After a 2 min isothermal hold at 60°C, the oven was temperature programmed to 290°C at 4°C/min with the final temperature held for 30.5 min. Acquisition was performed in selected ion monitoring (SIM) mode after a solvent delay of 6 min. The following *m/z* ions were selected to target terpanes and steranes characteristic of bitumen [23]: 83.10, 85.10, 113.10, 125.10, 133.10, 134.10, 142.10 177.20, 178.10, 183.20, 191.20, 192.20, 205.20, 217.20, 218.20, 221.20, 231.20 and 259.20.

G1701EA Chemstation (G1701EA) software was used for system control and data collection/manipulation.

Mass spectral data were interpreted manually with the aid of retention time data from previously analysed bitumen specimens, the NIST/EPA/NIH Mass Spectral Library version 2.0 and comparison with published data [24].

#### Isotopy

EA-IRMS analyses were performed using a Europa Scientific elemental analyser with 20–20 Europa Scientific IRMS. The measurements were performed on the asphaltene fraction of samples prepared by precipitation with DCM:Hexane (1:50) as described above. For hydrogen and carbon isotopes analysis amounts of 0.5 mg of asphaltene sample were sealed into combustion capsules (5 x 8 mm).

#### Carbon analysis

Tin capsules containing sample or reference material were placed into a furnace at 1000°C and combusted in an oxygen rich environment, raising the temperature in the region of the sample to c. 1700°C. The gases produced were swept in a helium stream over combustion catalyst (Cr_2_O_3_), copper oxide wires (to oxidize hydrocarbons) and silver wool (to remove sulphur and halides). The resultant gases were swept through a reduction stage of pure copper wires at 600°C (to remove O_2_ and convert NO_x_ species to N_2_) and a magnesium perchlorate chemical trap to remove water. Carbon dioxide was separated from nitrogen by a packed column gas chromatograph held at an isothermal temperature of 100°C, and then entered the ion source of the Europa Scientific 20–20 IRMS where it was ionised and accelerated. Species of different mass were separated in a magnetic field then simultaneously measured using a Faraday cup collector array to measure the isotopomers of CO_2_ at *m/z* 44, 45, and 46.

The reference material used for δ^13^C analysis was IA-R002 (mineral oil, δ^13^C_V-PDB_ = -28.06 ‰). IA-R002 has been calibrated against and is traceable to NBS-22 (mineral oil, δ^13^C_V-PDB_ = -29.81 ‰), an inter-laboratory comparison standard distributed by the International Atomic Energy Agency. Inter-laboratory comparison standard IA-R005 (beet sugar, δ^13^C_V-PDB_ = -28.06 ‰) and IAEA-CH-6 (sucrose, δ^13^C_V-PDB_ = -10.43 ‰, a sample calibrated against and traceable to IAEA-CH-6) were analysed alongside the samples as quality control checks.

#### Hydrogen analysis

Silver capsules containing sample or reference material were placed into a furnace at 1080°C and thermally decomposed to H_2_ and CO over glassy carbon. Trace water produced was removed by magnesium perchlorate and any traces of CO_2_ formed were removed via a Carbosorb^™^ trap. H_2_ was resolved by a packed column gas chromatograph held at 35°C. The resultant chromatographic peak entered the ion source of the IRMS where it was ionised and accelerated. Gas species of different mass were separated in a magnetic field then simultaneously measured on a Faraday cup universal collector array. For H_2_, masses 2 and 3 were monitored.

The reference material was IA-R002 (mineral oil, δ^2^H_V-SMOW_ = -111.2 ‰), calibrated against and traceable to NBS-22 (mineral oil, δ^2^H_V-SMOW_ = -118.5 ‰), an inter-laboratory comparison standard distributed by the International Atomic Energy Agency. Inter-laboratory comparison standard IAEA-CH-7 (polyethylene foil, δ^2^H_V-SMOW_ = -100.3 ‰) and IA-R062 (FIT-PTS 10/1/D olive oil, δ^2^H_V-SMOW_ = -137.06 ‰), an inter-laboratory proficiency testing scheme sample with a generally agreed δ^2^H value, were analysed alongside the samples as quality control checks.

## Results and Discussion

### Characterisation of the archaeological samples

Samples from the tarry lumps that had previously been characterised as Stockholm tar (BM Reg nos.: 1939.1010.250 and 1939.1010.251) were almost entirely soluble in DCM and the solvent-insoluble residue appeared to be minimal. In contrast the other black materials were almost entirely insoluble with negligible solvent-extractable content. No terpene compounds characteristic of conifer tars could be detected in any of the DCM extracts by GC-MS.

**Fig 4:**
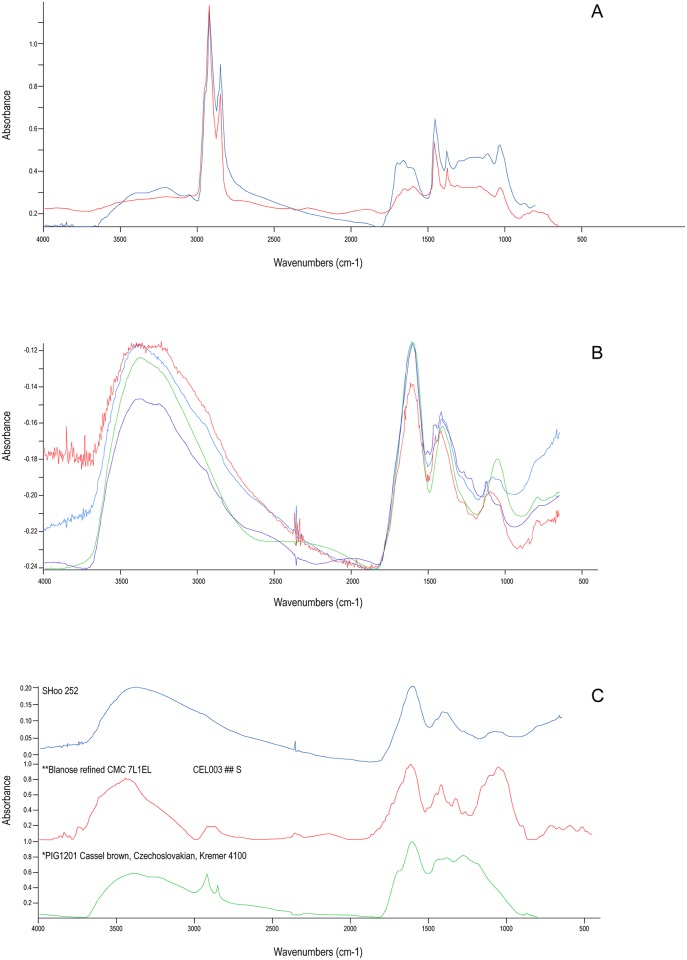
FTIR spectra obtained from tarry materials recovered from Mound 1, Sutton Hoo. (a) red spectrum = inv 251; blue spectrum = reference specimen of Dead Sea bitumen. (b) red spectrum = inv 255; blue spectrum = inv 252; green spectrum = inv 254; purple spectrum = inv 253. (c) Inv 252 (blue) with reference spectra of cellulose gel (Blanose CMC 7L1EL) and Cassel brown pigment (Kremer 4100).

The FTIR spectra of the putative tars 1939.1010.250 and 1939.1010.251 lacked the strong carbonyl (C = O str) band typical of pine-tar or tree resin, instead displaying less functionalised spectra, characteristic of bitumen (Fig 4a). Spectra obtained from the other tarry finds (1939.1010.252–5) were markedly different (Fig 4b), lacking the sharp C-H stretching bands at 3000–2800 cm^-1^ and they share some features with reference spectra of cellulose and to a lesser degree Cassel brown pigment (Fig 4c). The latter, as a bituminous earth, may indicate that these tar-like materials also have a fossil organic component, but the possibility that the spectra arise from contamination with soil-derived organic from the burial environment cannot be ruled out.

**Fig 5:**
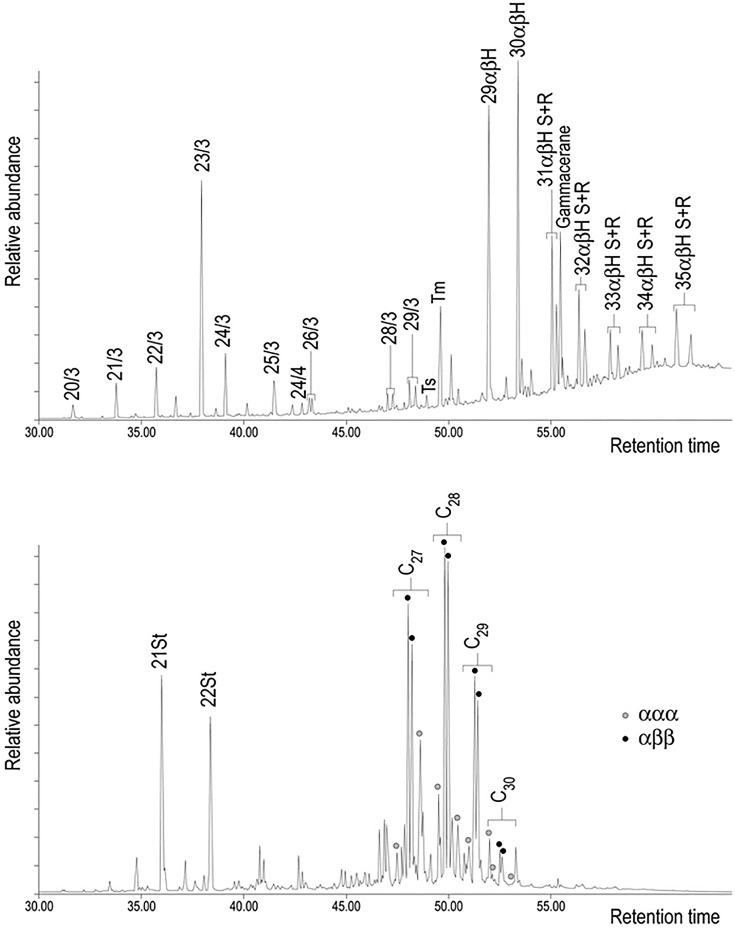
Mass chromatograms from Sutton Hoo sample 1939,1010.250 showing distribution of (a) terpanes (*m/z* 191) with *n*/3 [tricyclic terpane with *n* carbon atoms], *n*/4 [tetracyclic terpane with *n* carbon atoms], Tm [17α-22,29,30-trisnorhopane], Ts [18α-22,29,30-trisnorneohopane] and *n* αβH [17α,21β-hopanes with *n* carbon atoms in R and S configurations] and of (b) steranes with *n*St [short chain sterane with *n* carbon atoms] and C_n_ [long chain ααα- and αββ- steranes with *n* carbon atoms in R and S configurations].

Consistent with their solvent insolubility, samples 1939.1010.252–5 yielded no diagnostic organic compounds by GC-MS analysis so further characterisation of them is not possible without application of alternative analytical techniques. Further GC-MS analysis of fractionated extracts from fragments of 1939,1010.250 and 251, however, confirms the FTIR interpretation (Fig 5), revealing a series of tricyclic terpanes (*m/z* 191, C_19_-C_30_)_,_ a complete series of 17α,21β-hopanes (with C_35_ > C_34_ indicative of the maturity of the source rock) and a sterane distribution with short- and long-chain steranes (*m/z* 218, C_21_-C_22_ and C_27_-C_30_). These compounds are unambiguously fossil fuel biomarkers found in petroleum and archaeological bitumen [23, 25]. In fact, the bulk composition of the material displays all the characteristics of archaeological bitumen, containing low hydrocarbons (less than 10%) and high asphaltenes (60–85%; Table 1) [26]. It is thus clear that the Sutton Hoo residues were originally misidentified and are bituminous materials rather than conifer tars as hitherto supposed. In the course of this study, our research in the BM archives unearthed an unpublished scientific report dating to 1971 that challenged the original interpretation of Stockholm tar and proposed bitumen as an alternative based on reinterpretation of the original paper chromatography and new FTIR analyses, although the results of the latter were inconclusive. The revised interpretation came too late for inclusion in the publication of the finds and the earlier Stockholm tar attribution has continued to be cited in subsequent works and in the BM catalogue records [3, 17, 27–28]. As a consequence of this erroneous identification the significance of these materials in the burial has been both misunderstood and understated. Although bitumen has long been used to caulk boats in the Middle East [29–30], there is no evidence for its use in this context in Britain or northern Europe [31], even though local sources are available (Fig 1). The interpretation of the tarry lumps in the burial chamber as a mariner’s repair kit must now be rejected [3].

### Bitumen source

Fresh interpretation of these finds depends on determination of the origin of the bitumen. The most accessible native bitumen sources are located in the west of the British Isles. Although geographically close, cultural divisions in the early medieval period would have placed these outside the East Anglian domain and their appearance at Sutton Hoo would constitute important evidence for interactions between the East Anglian kingdom and the rest of the British Isles. It would also provide the first direct evidence for active exploitation of these native sources in antiquity and perhaps confirm a prosaic purpose for the material, allowing it to be counted among the other practical items in the burial assemblage. A distant source, in contrast, would constitute an apparently rare import of the material, more likely to be counted as a prestige item, to be grouped with the other luxury accoutrements.

To constrain its origin, the chemical composition of the Sutton Hoo bitumen was compared with a select group of British and Middle Eastern bitumens. Bitumen from Pitchford (Shropshire, UK) was selected because of its long history of exploitation: the location is recorded as “Piceforde” in the Domesday Book (*c*. 1086) [32] and the still extant bituminous well may have been used even earlier, as Romans at nearby Wroxeter are thought to have exploited local seeps [33]. Other localities considered, that did not yield sufficient biomarkers for correlation, include mines at Great Orme’s Head (Gwynedd, Wales) and South Crofty (Cornwall), where anciently exploited ore deposits co-occur with bitumen [34]. Specimens from three other on-shore petroleum systems (Windy Knoll, Derbyshire; Thurso, Caithness; Mupe Bay, Dorset) with substantial inland and coastal outcrops of vein-bitumen and bituminous sandstone were also included [35–36]. The Middle Eastern comparators are all known or reputed to be in active exploitation in the 1^st^ millennium AD and earlier (Table 2) [37].

**Table 2:** Gross composition and molecular ratios for Sutton Hoo and comparative bitumens.

		EOM (mg/g)	Saturated (%)	Aromatic (%)	Resin (%)	Asphaltene (%)	Ts : Tm	23/3: 24/4	GCR: 30αβH	% C27 St	% C28 St	% C29 St	Diasteranes	C30 *n*-propyl cholestane
*Sutton Hoo bitumen*	19,391,010.251	97.3	2	4	26	69	0.1	22.3	0.39	33	44	23	+	++
	19,391,010.251	nd	nd	nd	nd	nd	0.07	nd	0.56	39	38	23	+	++
	19,391,010.250	nd	nd	nd	nd	nd	0.06	nd	0.63	37	36	27	+	++
British bitumen sources*	Pitchford Bridge	77.8	17	20	38	26	1.29	3.79	0.05	35	23	42	++	++
	Row Brook	nd	nd	nd	nd	nd	0.99	5.01	0.13	35	29	36	++	++
	Snail Beach	nd	nd	nd	nd	nd	1.09	3.4	0.06	38	19	42	++	++
	Mupe Bay	nd	nd	nd	nd	nd	0.99	5.05	0.15	35	22	43	++	+
	Thurso	nd	nd	Nd	nd	nd	0.9	18.4	0.7	16	55	34	+	+
*Other ancient bitumen sources*	Albanian	nd	nd	nd	nd	nd	0.3	0.7	0.04	nm	nm	nm	++	+++
	Syrian	99.6	2	2.4	7.4	88.2	0.09	20.7	0.31	30.2	45	27.7	+	++
	Dead Sea	77	4	25	43	29	0.79	2.58	0.19	35	24	42	+	++
	Hasbeya asphalt †	99.1	1	11	20	69	0.13	4.48	0.16	41	34	25	+	++
	Dead Sea 69A2 †	nd	nd	nd	nd	nd	0.07	16.79	0.45	34	40	26	+	++
	Hit 231, (Iraq)†	na	na	na	na	na	0.1	0.5	0.23	32	24.1	44	na	na
	Hit 233, (Iraq)†	na	na	na	na	na	0.14	0.74	0.3	25.6	29.9	44.5	na	na
	Hit 232, (Iraq)†	na	na	na	na	na	0.13	0.75	0.23	30.6	24.3	45.1	na	na
	Hit 234, (Iraq)†	na	na	na	na	na	0.12	0.75	0.18	33	23.7	43.3	na	na
	Hit Abu Jir 135–1 (Iraq) †	na	na	na	na	na	0.11	0.53	0.14	na	na	na	na	na
	Hit Abu Jir135-2 (Iraq) †	na	na	na	na	na	0.13	0.57	0.15	36	21	43	na	na
	Hit 16 (Iraq) †	nd	nd	nd	nd	nd	0.18	0.68	0.97	29	25	46	na	na

EOM = extractable organic matter; Ts = C_27_ 17α-22,29,30-trisnorhopane;Ts = C_27_ 18α-22,29,30-trisnorneohopane; 23/3 = C_23_ 13β, 14α (H) tricyclic terpane; C24/4 = C_24_ 17,21-des-E-hopane (secohopane); GCR = gammacerane; 30αβH = C_30_ 17α, 21β(H) hopane; % C27 St, % C28 St, % C29 St = the relative percentage abundance of C_27_, C_28_, C_29_ 5α,14α,17α(H) 20S & 20R steranes; Diasteranes and C30 sterane = qualitative assessment of the abundance of diasteranes and C_30_ n-propylcholestane, + = present but not prominent on ion chromatograms, ++ = easily identified on ion chromatogram, +++ = prominent feature of ion chromatogram. nd = not done; nm = could not be accurately measured; na = information not available.

* Samples of bitumen from Great Orme (Llandudno, Wales), Windy Gnoll (Derbyshire, England), South Crofty, (Pool, Cornwall) were analysed but yielded biomarker data inadequate for correlation purposes.

^†^ data taken from reference [37].

The Sutton Hoo extracts appear richer in polar compounds and poorer in saturates relative to the native Pitchford bitumen, although this alone would not rule out a correlation. However, it is notable that its gross composition is very similar to published data from Middle Eastern bitumen of the geochemically well-described Dead Sea family [37–40].

More compelling evidence for a Middle Eastern origin comes from the asphaltene carbon stable isotope composition (δ^13^C) and petroleum biomarker parameters. These are considered reliable indicators for the origin of archaeological bitumen providing they have not been severely affected by weathering, which can modify gross and molecular compositions [30, 41]. Hydrogen isotopic values (δD) on the other hand are sensitive to these alteration phenomena, leading to enrichment in deuterium in archaeological samples [30, 37, 41]. Isotopic values of the asphaltene fraction recovered from Sutton Hoo sample 1939,1010.251 (δ^13^C: -29.2 ‰ and δD: -100.9 ‰) are consistent with other published asphaltene isotopic values obtained for archaeological Dead Sea bitumen [37, 41, 42, 43].

Terpane and sterane patterns provide the key molecular parameters that are routinely used to determine the geochemical source of archaeological bitumen (see for example: [29, 44]). The Sutton Hoo bitumen is characterised by a regular and almost complete series of tricyclic terpanes (C_19_-C_30_), maximising at C_23_ and a complete series of 17α,21β-hopanes (Fig 3). The specific distribution of 17α,21β-hopanes with C_35_ > C_34_ is indicative of the maturity of the source rock [45]. The Sutton Hoo sterane distribution is dominated by regular steranes, that is to say short chain steranes (abbreviated as C_21_St and C_22_St in the figures) and C_27_-C_30_ steranes maximising at C_28_.

The relative distribution of certain petroleum-biomarkers provides a strong qualitative link between Dead Sea [29, 44] and Sutton Hoo bitumen (Table 2); namely a low abundance of diasteranes and tetracylic terpanes and relatively high abundances of C_30_ *n*-propylcholestanes. The absence and respective presence of these biomarkers provides a fundamental fingerprinting characteristic, imparted to the bitumen during its formation from precursor fossil organic matter [46–48], and strongly links the Sutton Hoo bitumen with Middle Eastern sources [29, 44], as opposed to the other largely British sources listed in Table 1.

**Fig 6:**
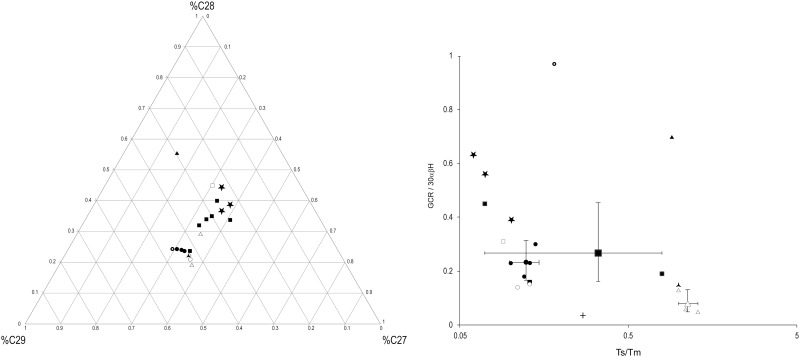
a) Ternary diagram showing distribution of long chain steranes as % C_27_ St (C_27_ αββsteranes R and S), % C_28_ St (C_28_ αββSteranes R and S), % C_29_ St (C_29_ αββsteranes R and S) [% C_27_ St _=_ C_27_ St / C_27_ St + C_28_ St + C_29_ St]. b) cross plot of Ts/Tm and GCR/30αβH. Tm = C27 17α-22,29,30-trisnorhopane; Ts = C27 18α-22,29,30-trisnorneohopane; GCR = gammacerane; 30αβH = C30 17α, 21β(H) hopane. Sutton Hoo bitumen (★), British bitumen from Shropshire (△), Mupe Bay (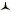) and Thurso (▲); Dead Sea (■); Syrian (□); Hit, Iraq (●); Hit 16, Iraq (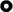);Hit-Abu Jir, Iraq (○). For source data see Table 2. The larger symbols and tie lines correspond to average values and their range.

Graphs of selected molecular ratios representative of the discriminating characteristics of terpanes and regular steranes are shown in Fig 6. The ratios of Ts (C_27_ 18α-22,29,30-trisnorneohopane) to Tm (C_27_ 17α-22,29,30-trisnorhopane) and gammacerane to C_30_ 17α,21β (H) hopane have previously been employed as reliable genetic parameters for determining the sources of archaeological bitumen [30, 37, 42]. Based on these graphs the strongest affiliation for the Sutton Hoo bitumen is with the Dead Sea and not British bitumen. Furthermore, abundant tricyclic terpanes together with a high concentration of gammacerane (as observed in the Sutton Hoo samples) have been described as typical characteristics of the Dead Sea family [37], whereas a lack of tricyclic terpanes, slightly higher Ts to Tm ratio and C_27_-C_29_ steranes maximising at C_29_ are characteristic of bitumen from the deposit of Hit-Ramadi-Abu Jir, Iraq, a major source of bitumen along the Euphrates river which was extensively exploited and exported in antiquity [23, 29, 49].

The ternary diagram presented in Fig 6a represents the gross composition of regular steranes. The Pitchford bitumen together with other British bitumen analysed, cluster separately from the well-differentiated Middle Eastern major asphalt sources, the Dead Sea family and the Hit-Ramadi-Abu Jir deposit respectively [37]. A strong similarity between the gross composition of the regular steranes of the Sutton Hoo and Dead Sea family bitumen can rather be seen (Fig 6a) and the Sutton Hoo terpane and sterane patterns have their most striking molecular analogies with those from geologically oil-stained rocks found 15 km north of Umm El Tlel and from the Jebel Bichri, both located in Syria [29]. Whether the Sutton Hoo bitumen originates from one of these seepages or from another comparable source in the same area, yet to be found, including possibly sources that have since disappeared as a result of erosion or human over-exploitation, remains unclear. A larger comparator sample base would be needed to confirm a connection with a Syrian-bitumen family as above the more general Dead Sea family of bitumen. The potential impact of more than a thousand years in the acidic burial environment of Sutton Hoo should also be considered before closer parallels can be drawn with more specific sources. Alternative sources within the Eurasian continent might also be considered; bitumen trade between the Black Sea and the Mediterranean is archaeologically attested in earlier periods [50] and although the source of this material is not proven its location on the inland river routes linking to the Baltic and North Seas may be significant. What is clear is that the Sutton Hoo bitumen does not correlate to any of the British petroleum systems investigated in this study.

### Archaeological significance

A Levantine origin for the Sutton Hoo bitumen demands a fresh interpretation of the finds and their significance. Despite the widespread occurrence of accessible seeps and outcrops within the British Isles, archaeological finds of bitumen in Britain are rare: two Roman cinerary urns (from Sussex and Kent) have been described as bitumen coated [51] but the only analytically confirmed bitumen is on an Iron Age sword from Yorkshire [52]. Whether these rare examples derive from the native sources or are indicative of importation from afar is unknown as none has been geochemically sourced.

The novelty of bitumen in the region could be a factor behind its appearance at Sutton Hoo. From its location on the eastern seaboard the East Anglian kingdom faced east across the North Sea to engage more closely with continental Europe and Mediterranean contacts beyond than with the western British Isles where native bitumen is most accessible [53]. The Levantine bitumen source accords with the more distant provenance of other objects in the burial assemblage (Fig 1). Among these are Levantine textiles, a ‘Coptic’ bowl and Imperial table silver from Byzantium [3, 17, 54].

These objects sit within wider evidence in East Anglia for continental contact and influence during this period, attested by archaeological finds such as decorative metal fittings, jewellery and pottery [55]. Imported coinage from across the continent, including Byzantine and even Sasanian coins, is also evident [56]. The mechanisms by which such goods arrived are complex to read and may include migration, settlement and trade as well as prestige gifts or diplomatic gestures between the elites of Europe [55; 57]. Trade routes can be inferred to have operated across the North Sea connecting to southern Europe and Mediterranean *via* the Rhine valley, along the Atlantic seaboard from the English Channel and *via* the Baltic and the Dneper to the Black Sea. The former was probably the most important route for the Sutton Hoo elite on the evidence of the coinage from the burial [56] and geographic convenience [58]. The discovery of Levantine bitumen at Sutton Hoo adds a new source of evidence for this transcontinental network of contacts.

**Fig 7:**
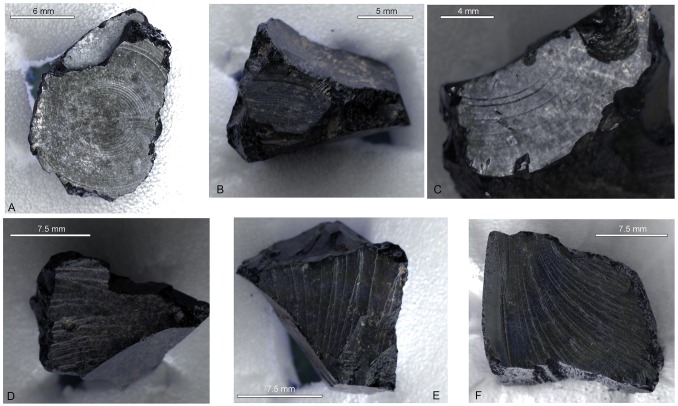
RTI images of surface morphology on fragments from 1939,1010.251. Upper images (A, B and C) show concentric rings suggestive of working or worked imprints; lower images show natural fracture surface on the same fragments (D and E) and on a reference specimen of bitumen (F).

Material thus acquired is unlikely to have been viewed in a utilitarian perspective, despite the usefulness of bitumen as a general purpose sealant and multi-purpose adhesive, for example, to repair ceramics [23, 59]. However, these same properties mean that bitumen can be used to fabricate composite objects [60] and mount precious stones and metals [29]. The faint concentric striations visible on some of the fragments were noted (in correspondence) when the objects were submitted to the BM Research Laboratory for analysis in 1969 with the question of whether these might be ‘the impress of a finely turned object’ or evidence for working of the material itself into an artefact. Closer examination of these surfaces using RTI and optical microscopy (Fig 7) does not allow a firm conclusion to be drawn but compared with the morphology of natural conchoidal fractures on the same fragments and on reference specimens the lines appear more even and concentric and more closely spaced. If the fragments were components of other burial objects, no such association was noticed at the time of excavation, although none could be expected if they were components of perishable (e.g. wooden) objects that did not survive. Bruce-Mitford, corresponding with the BM Research Laboratory in 1970 refers to ‘Stockholm tar … actually adhering to leather from the shield’ but on the basis of location this must be associated with the inv. 252 group of black organic fragments, the identity of which remains elusive.

Bitumen can, of course, also be moulded to manufacture ornamental items such as beads, dice and gaming pieces [29, 61, 62] and the lumps from Sutton Hoo may be the fragmentary remains of small bitumen objects of this kind. Their distribution at the head and foot of the coffin places them close to the areas where the ivory gaming pieces in the burial were discovered, but the locations do not correlate well enough to infer an association: most of the ivory fragments were found near the shield and other objects along the west wall of the chamber, while the best preserved ivory gaming piece was found underneath the silver dish. The highly fractured fragments do not offer any morphological evidence from which an original shape can be inferred, although a file note in the British Museum archive quotes an extract from C.W. Phillips’ diary recording the excavation of some of the pieces (1939.1010.251) on Tuesday July 25^th^ 1939: ‘*Some pieces of a broken lump of black material with a conchoidal fracture which had lain near* [the pottery bottle] *were also removed*’. This seems to suggest that these pieces were part of a single larger whole and also perhaps that not all of the fragments were recovered.

Small, highly valuable, portable items are typical in grave assemblages and, if the apparent rarity of bitumen in the British archaeological record is a reliable reflection of its past availability, the exotic quality of the material could have added further to the prestige of such items. Alternatively, it is possible that amorphous specimens may have been valued sufficiently to be placed in the burial chamber for their material novelty or other properties, such as medicinal use [63, 64].

Few clues as to the form of the bitumen can be gleaned from contemporary finds in the Near East. The wealth of reported archaeological bitumen finds in the region come from earlier millennia [29, 43] although the use of bitumen as an incendiary device in warfare by the Byzantine Empire (renowned as ‘Greek Fire’) is well- reported by the documentary sources [65]. The profitable export of lump Dead Sea bitumen from Eastern Mediterranean ports that was underway from the 12^th^ century BC [66] highlights the possibility that as an exported raw material bitumen could have been used in the production of objects in any location it passed through on route northwards.

Although the form of the bitumen fragments when they entered the burial cannot be interpreted with confidence, it is very clear that they should now be counted among the grave goods, either as components of fabricated objects or prized objects in their own right.

## Conclusions

This new multi-analytical study of black organic lumps discovered in the burial chamber of Mound 1 at Sutton Hoo, using FTIR, GC-MS and EA-IRMS, has overturned previous interpretation of the material as Stockholm tar and demonstrated instead that the fragments are composed of bitumen. The molecular and isotopic signatures of the bitumen suggest a Middle Eastern source rather than local origin in the UK. Archaeological finds of bitumen in Britain are very rare and this study presents the first material evidence for trading of the Middle Eastern bitumen northwards to reach the British Isles.

This fresh characterisation, coupled with the distribution of the bitumen in the burial chamber, indicates that the bitumen should be interpreted as part of the grave goods rather than related to the construction of the ship. The possible Syrian origin of the bitumen is particularly interesting given that other items in the burial assemblage have been linked to this region. Nevertheless, the significance of the bitumen lumps among the grave goods is not clear as their morphology offers little evidence for their original form: possibly they represent surviving components of perishable objects, fragmentary small bitumen objects or, alternatively, the material may have been valued in its own right as a prestige raw material.
